# Changes in Dementia-Related Behavioral Symptoms Observed by Hospice Staff During COVID-19

**DOI:** 10.1093/geroni/igab046.1081

**Published:** 2021-12-17

**Authors:** Kimberly Convery, Tessa Jones, Aditi Durga, Abraham Brody, Shih-Yin Lin

**Affiliations:** 1 New York University Rory Meyers College of Nursing, New York, New York, United States; 2 New York University Silver School of Social Work, New York, New York, United States; 3 NYU Rory Meyers College of Nursing, New York, New York, United States; 4 NYU Hartford Institute for Geriatric Nursing, New York, New York, United States; 5 New York University, New York, New York, United States

## Abstract

COVID-19 infection control precautions (e.g., social distancing) and associated isolation and changes to routines can worsen dementia-related behavioral symptoms. A cross-sectional online survey was administered to 101 hospice staff (95% female; mean age 49) to investigate what dementia-related behavioral symptoms in their care recipients had changed from before to after the COVID-19 outbreak. Of the 101 participants, 47 (46.5%) reported changes in symptoms, three (3%) had not been able to physically observe/assess their care recipients, two (2%) reported changes in routines, and 49 (48.5%) reported no changes. The most common changes in symptoms were increased agitation (N=19), depression (N=16), confusion (N=10), and anxiety (N=6). Some participants (N=14) also commented on potential causes, including not having visitors and inability to go outside for normal activities while not understanding why. Interventions to normalize social environments for hospice recipients with dementia are likely imperative to alleviate behavioral symptoms exacerbated by COVID-19-related precautions.

